# Racial and ethnic disparities in neoadjuvant chemotherapy patterns and outcomes in early-stage HER2-positive breast cancer

**DOI:** 10.1038/s41523-025-00854-4

**Published:** 2025-12-05

**Authors:** Inimfon Jackson, Xiudong Lei, Hui Zhao, Rashmi Murthy, Sharon H. Giordano, Mariana Chavez-MacGregor

**Affiliations:** 1 Division of Cancer Medicine, The University of Texas MD Anderson Cancer Center, Houston, TX USA; 2 Department of Health Services Research, The University of Texas MD Anderson Cancer Center, Houston, TX USA; 3 Department of Breast Medical Oncology, Division of Cancer Medicine, The University of Texas MD Anderson Cancer Center, Houston, TX USA

**Keywords:** Cancer, Health care, Oncology

## Abstract

We examined neoadjuvant chemotherapy (NACT) use, pathological complete response (pCR) and the association with overall survival (OS) among patients with early-stage HER2-positive breast cancer (BC). Patients ≥18 years with stage I-III HER2-positive BC from 2010–2022 who had surgery and chemotherapy were identified. Of 195,023 patients treated with chemotherapy, 37.7% received NACT. NACT use increased from 18.6% in 2010 to 63.4% in 2022 (*p* < 0.001) and pCR rates rose from 21% to 47.6% (*p* < 0.001). Black patients were less likely to receive NACT (aOR = 0.96;95%CI 0.93–0.99) or achieve pCR (aOR = 0.86;95%CI 0.82–0.90) than White patients. pCR was associated with a reduction in the risk of death (aHR = 0.45;95%CI 0.42–0.48). 3-year OS increased from 91% in 2010 to 95% in 2019 for patients without a pCR (*p* < 0.001), and from 97% to 99% for patients with pCR (*p* = 0.002). Further research is needed to understand and address racial and ethnic disparities in treatment access and outcomes.

## Introduction

Approximately 20% of all breast cancers have an amplification of the human epidermal growth factor receptor 2 (HER2/neu) gene, which plays an important role in tumor proliferation and progression of disease^1–3^. HER2-positive breast cancer (BC) is clinically and pathologically aggressive and prior to the development of HER2-targeted therapies, was associated with poorer outcomes^4,5^. Neoadjuvant chemotherapy (NACT) has been shown to be beneficial in locoregional tumor downstaging, increasing breast conservation rates and allowing for de-escalation of axillary surgery^6–9^. Additionally, NACT enables the individualization of adjuvant treatment based on the presence or absence of pathological complete response (pCR) allowing for escalation of therapy according to the in vivo response of the tumor^1,6,10^. International guidelines recommend the use of NACT for HER2-positive BCs measuring ≥2 cm or with lymph node involvement (≥cN1)^6,11,12^. In these settings, NACT combined with anti-HER2 therapy is the standard of care, associated with increased pCR rates and significantly improved survival outcomes^12^.

Research conducted on the trends in NACT use demonstrates an increase over time^13–19^. A study showed that among patients with stage I HER2-positive BC there was an increase in use from 4.2% in 2010 to 17.3% in 2015^18^. Analysis of data from women diagnosed with stage I to IIIA BC at Kaiser Permanente Northern California and Washington databases reported an increasing trend in NACT use across BC subtypes, overall, and specifically among those with HER2-positive tumors^19^. However, these patterns have not been assessed across different clinical cancer stages or by pCR status. Furthermore, racial and ethnic disparities in patterns of use of NACT and associated outcomes remain understudied. We assessed the treatment patterns and trends of NACT use, subsequent pCR and OS, and examined the factors associated with NACT use and pCR in a large contemporary database of patients with localized HER2-positive BC.

## Results

### Baseline Characteristics

195,023 patients with stage I-III HER2-positive BC who received chemotherapy were included, of them, 37.75% received NACT and the rest adjuvant chemotherapy. The median age at diagnosis was 56 years (IQR: 47–65 years). The majority of the HER2-positive BC patients were non-Hispanic White (subsequently referred to as White) (73.1%), with HR-positive disease (70.6%), no comorbidity (85.1%), residing in metropolitan areas (82%) and were treated with combination chemotherapy (79.5%). Furthermore, 61.4% of the patients had private insurance. Overall, 48.2%, 38.9%, and 12.8% of the patients had clinical stage I, II, and III disease, respectively (Table 1).

**Table 1:** Baseline characteristics of patients diagnosed with HER2-positive BC (2010–2022) who were treated with chemotherapy and surgery in the NCDB database (*N* = 195,023)

Covariates	Total *N* (Column %)	Adjuvant chemotherapy *N* (Column %)	Neoadjuvant chemotherapy *N* (Column %)	*P*
*N*	195023 (100)	121396 (100)	73627 (100)	
Year of diagnosis				
2010–2013	49338 (25.3)	39067 (32.2)	10271 (14)	<0.001
2014–2017	67991 (34.9)	43229 (35.6)	24762 (33.6)	
2018–2022	77694 (39.8)	39100 (32.2)	38594 (52.4)	
Age at diagnosis (years, median)	56	57	54	
18–49	60438 (31)	33061 (27.2)	27377 (37.2)	<0.001
50–59	56607 (29)	35103 (28.9)	21504 (29.2)	
60–64	27000 (13.8)	17710 (14.6)	9290 (12.6)	
65–74	38097 (19.5)	26446 (21.8)	11651 (15.8)	
≥75	12881 (6.6)	9076 (7.5)	3805 (5.2)	
Race and ethnicity				
Black	24253 (12.4)	14911 (12.3)	9342 (12.7)	<0.001
Hispanic	14264 (7.3)	7698 (6.3)	6566 (8.9)	
White Non-Hispanic	142490 (73.1)	90916 (74.9)	51574 (70)	
Other	14016 (7.2)	7871 (6.5)	6145 (8.3)	
Hormone receptor status				
Negative	57395 (29.4)	33001 (27.2)	24394 (33.1)	<0.001
Positive	137628 (70.6)	88395 (72.8)	49233 (66.9)	
AJCC clinical stage				
I	94060 (48.2)	71442 (58.9)	22618 (30.7)	<0.001
II	75906 (38.9)	39331 (32.4)	36575 (49.7)	
III	25057 (12.8)	10623 (8.8)	14434 (19.6)	
AJCC clinical tumor stage				
T0	348 (0.2)	234 (0.2)	114 (0.2)	<0.001
T1	88741 (45.5)	72510 (59.7)	16231 (22)	
T2	78521 (40.3)	38142 (31.4)	40379 (54.8)	
T3	17800 (9.1)	6989 (5.8)	10811 (14.7)	
T4	8781 (4.5)	2922 (2.4)	5859 (8)	
Unknown	832 (0.4)	599 (0.5)	233 (0.3)	
AJCC clinical nodal status				
N0	131425 (67.4)	89988 (74.1)	41437 (56.3)	<0.001
N1	51241 (26.3)	25333 (20.9)	25908 (35.2)	
N2	6395 (3.3)	3050 (2.5)	3345 (4.5)	
N3	4776 (2.4)	2093 (1.7)	2683 (3.6)	
Unknown	1186 (0.6)	932 (0.8)	254 (0.3)	
Comorbidity				
0	166028 (85.1)	102104 (84.1)	63924 (86.8)	<0.001
1	22452 (11.5)	14927 (12.3)	7525 (10.2)	
2+	6543 (3.4)	4365 (3.6)	2178 (3)	
Chemotherapy				
Combination	155129 (79.5)	89854 (74)	65275 (88.7)	<0.001
Single	35929 (18.4)	28916 (23.8)	7013 (9.5)	
Unknown	3965 (2)	2626 (2.2)	1339 (1.8)	
Insurance				
Private	119791 (61.4)	72504 (59.7)	47287 (64.2)	<0.001
Medicaid	17015 (8.7)	9589 (7.9)	7426 (10.1)	
Medicare	49581 (25.4)	34390 (28.3)	15191 (20.6)	
Other government insurance	2488 (1.3)	1468 (1.2)	1020 (1.4)	
No insurance	4298 (2.2)	2359 (1.9)	1939 (2.6)	
Unknown	1850 (0.9)	1086 (0.9)	764 (1)	
Education quartile				
1 least educated	29656 (15.2)	18129 (14.9)	11527 (15.7)	<0.001
2	40361 (20.7)	25458 (21)	14903 (20.2)	
3	48902 (25.1)	30929 (25.5)	17973 (24.4)	
4	48726 (25)	30329 (25)	18397 (25)	
Unknown	27378 (14)	16551 (13.6)	10827 (14.7)	
Income quartile				
1^st^ Quartile	25468 (13.1)	16161 (13.3)	9307 (12.6)	<0.001
2^nd^ Quartile	33351 (17.1)	21124 (17.4)	12227 (16.6)	
3^rd^ Quartile	40032 (20.5)	25119 (20.7)	14913 (20.3)	
4^th^ Quartile (higher)	68758 (35.3)	42417 (34.9)	26341 (35.8)	
Unknown	27414 (14.1)	16575 (13.7)	10839 (14.7)	
Area of residence^a^				
Metro	159849 (82)	98833 (81.4)	61016 (82.9)	<0.001
Urban	24656 (12.6)	15938 (13.1)	8718 (11.8)	
Rural	3832 (2)	2440 (2)	1392 (1.9)	
Unknown	6686 (3.4)	4185 (3.4)	2501 (3.4)	
Region				
South	65908 (33.8)	41620 (34.3)	24288 (33)	<0.001
Northeast	34761 (17.8)	23573 (19.4)	11188 (15.2)	
Midwest	43916 (22.5)	27942 (23)	15974 (21.7)	
West	30187 (15.5)	18235 (15)	11952 (16.2)	
Unknown	20251 (10.4)	10026 (8.3)	10225 (13.9)	
Facility Type				
Community Cancer Program (CCP)	70511 (36.2)	45871 (37.8)	24640 (33.5)	<0.001
Comprehensive CCP	12053 (6.2)	8004 (6.6)	4049 (5.5)	
Academic	54798 (28.1)	34287 (28.2)	20511 (27.9)	
Integrated Network	37410 (19.2)	23208 (19.1)	14202 (19.3)	
Unknown	20251 (10.4)	10026 (8.3)	10225 (13.9)	

*AJCC* American Joint Committee on Cancer, *CCP* Community Cancer Care Program.

^a^Estimated by matching the state/county Federal Information Processing Standards code of the patient at diagnosis to 2013 data published by the US Department of Agriculture Economic Research Service. Metropolitan counties are defined as having a population size of the metropolitan area greater than 250 000. Urban counties are defined as non-metropolitan with a population size of at least 2500. Rural counties have a population of fewer than 2500.

### Trends and factors associated with NACT use

**Fig. 1:**
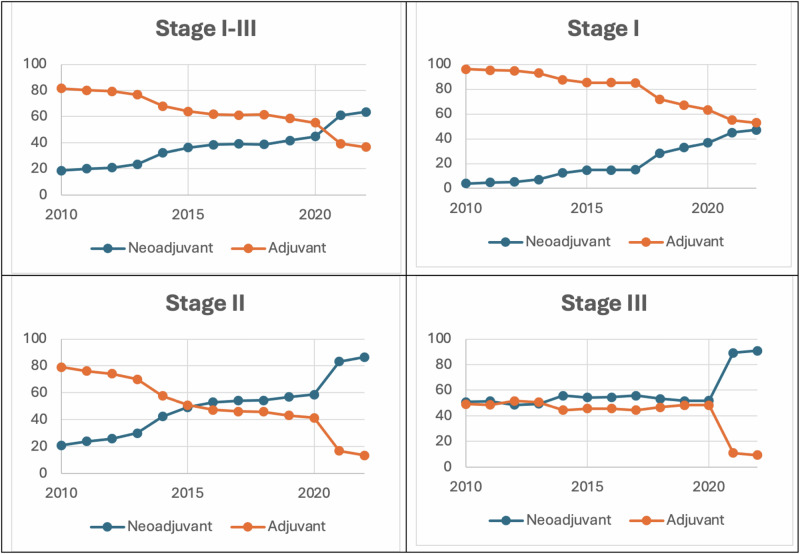
Rates of chemotherapy (neoadjuvant or adjuvant) among patients with HER-positive breast cancer stratified by clinical stage.

**Fig. 2:**
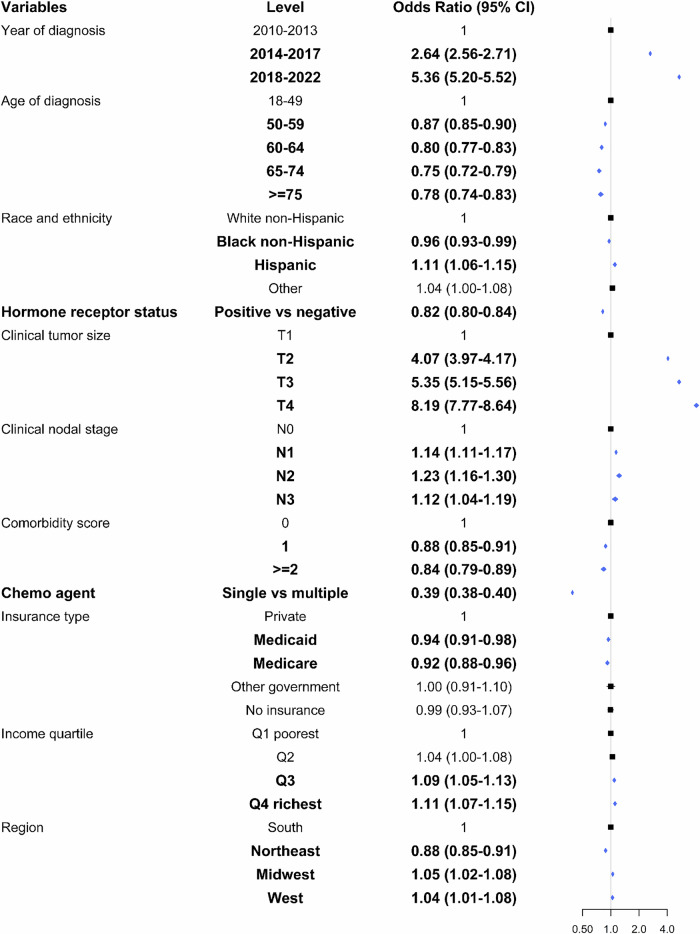
Multivariable logistic regression model for neoadjuvant chemotherapy use among patients with HER2-positive BC who underwent chemotherapy (*n* = 195,023). Note: The multivariate model was additionally adjusted for area of residence and facility type.

The rate of NACT use increased from 18.6% in 2010 to 44.8% in 2020, and 63.4% in 2022 (*p* < 0.001). When stratified by AJCC clinical stage, NACT receipt increased from 3.9% to 47.1%, 20.9% to 86.5% and 50.9% to 90.7% between 2010 and 2022 among patients with clinical stage I, II and III disease, respectively (Fig. 1). On multivariable logistic regression analysis, patients who underwent chemotherapy were more likely to receive NACT in both the 2014–2017 (aOR = 2.64; 95%CI 2.56–2.71) and 2018–2022 (aOR = 5.36; 95%CI 5.20–5.52) periods compared to the 2010–2013 period. Black patients had lower odds (aOR = 0.96; 95%CI 0.93–0.99) while Hispanic patients had higher odds (aOR = 1.11; 95%CI 1.06–1.15) of NACT use relative to White patients. Notably, patients with HR positive disease (aOR = 0.82; 95%CI 0.80–0.84) were less likely to receive NACT compared to those with HR negative status. Other factors associated with greater odds of NACT receipt included higher clinical tumor sizes and nodal stages, and higher income quartiles. Older age at diagnosis, greater comorbidity scores and having Medicaid/Medicare insurance was associated with lower odds of NACT use (Fig. 2).

### Trends and factors associated with pCR

**Fig. 3:**
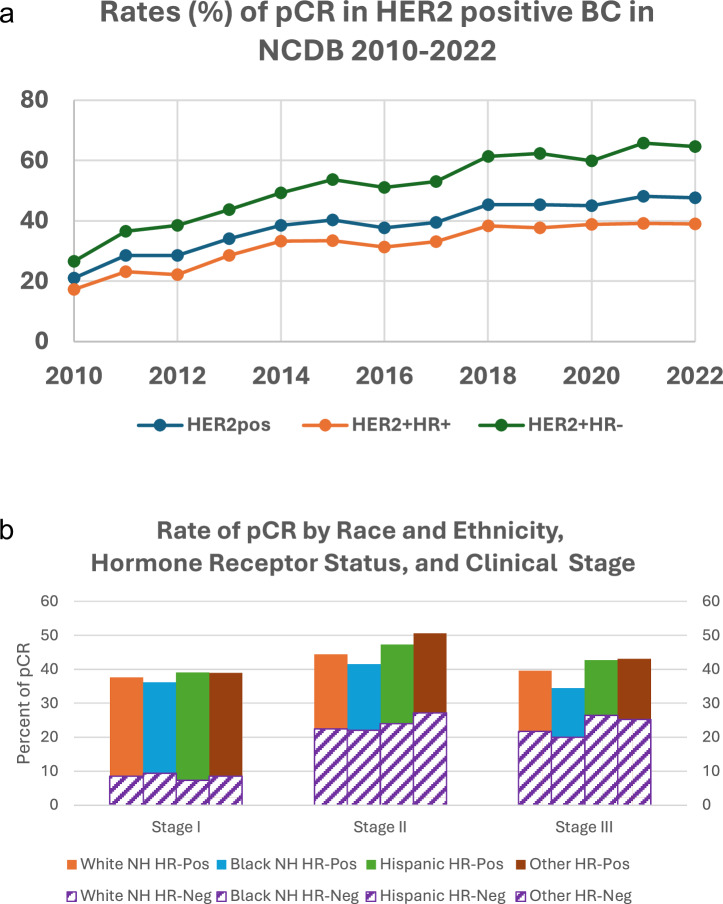
Rates of pathologic complete response (pCR) among patients with early-stage HER2-positive breast cancer who received neoadjuvant chemotherapy. **a** Rates of pathologic complete response (pCR) among patients with HER2-positive breast cancer who underwent neoadjuvant chemotherapy according to hormone receptor status. **b** Rates of pathologic complete response (pCR) among patients with HER2-positive breast cancer treated with neoadjuvant chemotherapy according to race and ethnicity, hormone receptor status and clinical stage.

**Fig. 4:**
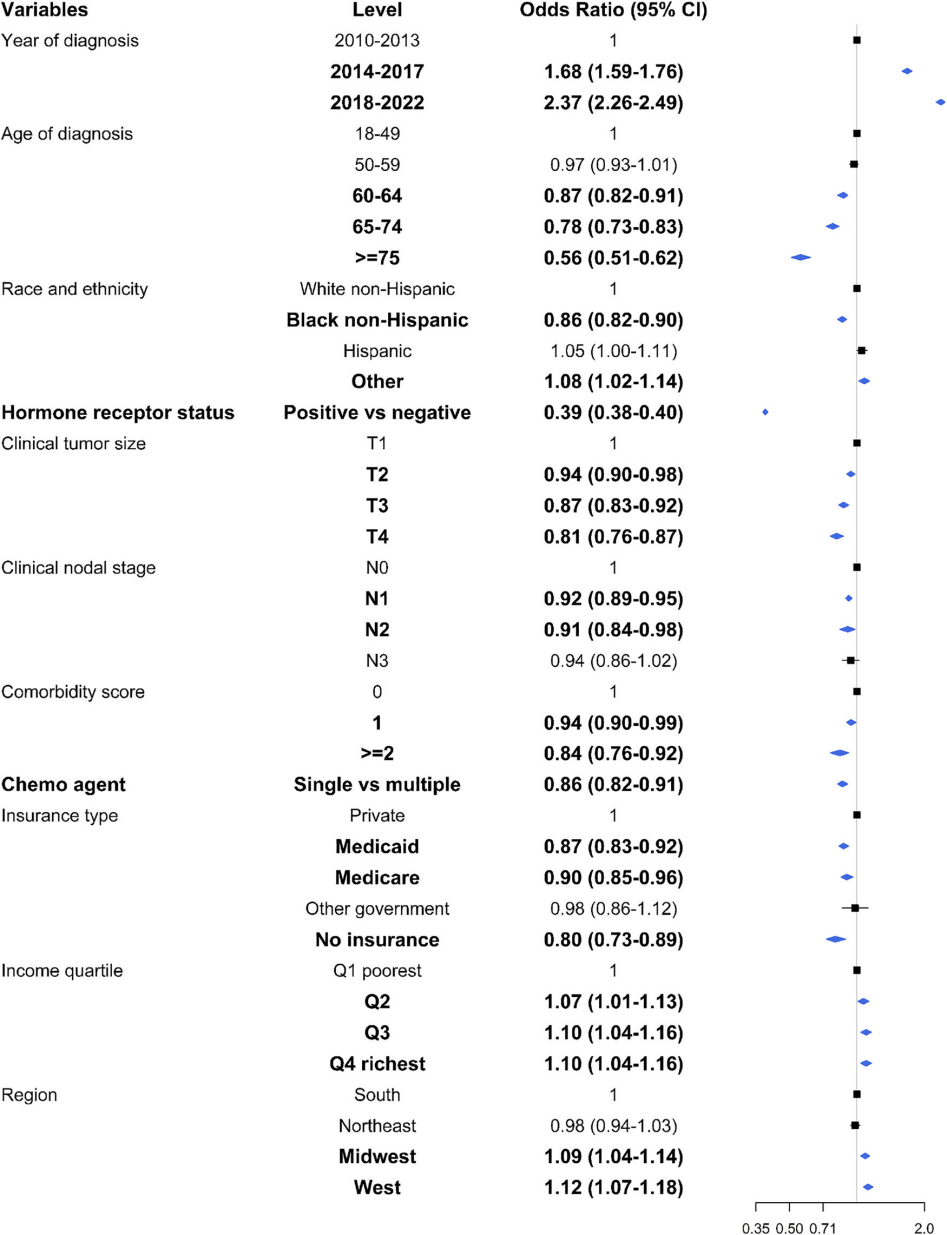
Multivariable logistic regression model for pathologic complete response (pCR) among patients with HER2-positive breast cancer who received neoadjuvant chemotherapy (*n* = 73,627).

Among the 73,627 patients with HER2-positive BC who received NACT, 41.5% achieved a pCR (Supplementary Table 2). Overall, pCR rates increased significantly from 21% in 2010 to 47.6% in 2022 (*p* < 0.001). Among patients with HR-positive disease, pCR rates rose from 17.3% in 2010 to 39% in 2022 (*p* < 0.001), while in those with HR-negative disease, rates increased from 26.6% in 2010 to 64.6% in 2022 (*p* < 0.001) (Fig. 3a). Notably, pCR rates varied by race and ethnicity. Hispanic patients had consistently higher pCR rates across clinical cancer stages, while Black patients had the lowest. Among patients with stage III BC, pCR was achieved in 42.7% of Hispanic patients, 39.5% of White patients, and only 34.5% of Black patients (Fig. 3b). After multivariable analysis, patients who received NACT between 2014–2017 (aOR = 1.68; 95%CI 1.59–1.76) and 2018–2022 (aOR = 2.37; 95%CI 2.26–2.49) were more likely to achieve a pCR compared to those who had NACT from 2010–2013. Relative to White patients, Black patients (aOR = 0.86; 95%CI 0.82–0.90) had lower odds of achieving a pCR while Hispanic patients (aOR = 1.05; 95%CI 1.00–1.11) had similar odds. Furthermore, patients with HR-positive tumors (aOR = 0.39; 95%CI 0.38–0.40) were less likely to have pCR compared to those with HR-negative status. Compared to clinical tumor size T1, patients with T2 (aOR = 0.94; 95%CI 0.90–0.98), T3 (aOR = 0.87; 95%CI 0.83–0.92), or T4 (aOR = 0.81; 95%CI 0.76–0.87) had lower likelihood of pCR. Other factors associated with decreased odds of pCR included higher clinical nodal stage, greater comorbidity scores, and Medicaid/Medicare or no insurance coverage (Fig. 4).

### Impact of pCR on OS

**Fig. 5:**
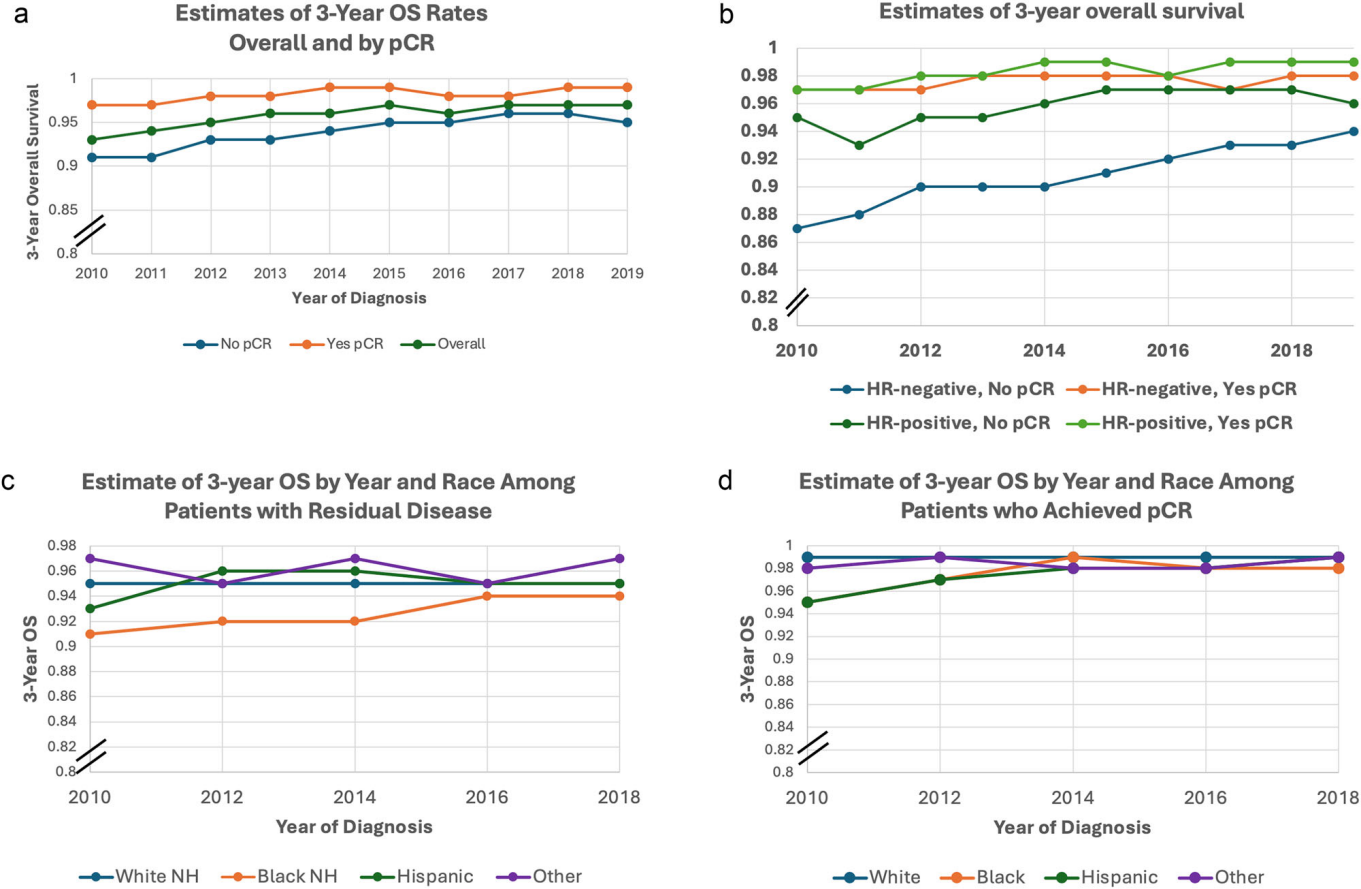
Estimates of 3-year overall survival rates. **a** Estimates of 3-year overall survival rates overall and according to pathological complete response (pCR). **b** Estimates of 3-year overall survival by year of diagnosis according to hormone receptor status, and pCR. **c** Estimates of 3-year overall survival by year of diagnosis according to race and ethnicity among patients with residual disease. **d** Estimates of 3-year overall survival by year of diagnosis according to race and ethnicity among patients who achieved pCR.

The median follow-up was 5.1 years (range 0.2–14.1 years). Overall, the 3-year OS was 98% among patients who achieved a pCR and 94% among those who did not (*p* < 0.001). In the overall population, the 3-year OS increased from 91% in 2010 to 95% in 2019 among those who did not achieve a pCR (*p* < 0.001), and from 97% to 99% in those with pCR (*p* = 0.002) (Fig. 5a). Among patients with HR-negative tumors, 3-year OS increased from 87% in 2010 to 94% in 2019 among those with no pCR (*p* < 0.001) and from 97% to 98% among those with a pCR (*p* = 0.02). Similarly, among those with HR-positive tumors, the 3-year OS increased from 95% in 2010 to 96% in 2019 among patients with no pCR (*p* = 0.02) and from 97% in 2010 to 99% in 2019 in those with a pCR (*p* = 0.005) (Fig. 5b). When stratified according to race and ethnicity among patients with residual disease, Black patients persistently had the lowest 3-year OS rates (Fig. 5c). However, among patients with pCR, 3-year OS was high across all groups. In 2010, Black and Hispanic patients both had 3-year OS rates of 95%, which improved to 98% and 99%, respectively, by 2018 (Fig.5d). After adjusting for possible confounders and propensity score, having a pCR (aHR = 0.45; 95% CI 0.42–0.48) was associated with a lower risk of death when compared to no pCR. Compared to White patients, Black patients (aHR = 1.14; 95%CI 1.06–1.22) had a higher risk of death while Hispanic patients (aHR = 0.82; 95%CI 0.73–0.92) had a lower risk of death (Table 2).

**Table 2:** Multivariable Cox proportional hazards model for overall survival among patients with HER2-positive BC who received neoadjuvant chemotherapy with propensity score adjustment (n = 64,203)

Covariates	Hazard Ratio	95% CI	*P*
pCR: yes vs no	0.45	0.42–0.48	<0.001
Age at diagnosis (ref. ^6,18–47^)	1		
50–59	1.25	1.15–1.35	<0.001
60–64	1.32	1.20–1.46	<0.001
65–74	1.55	1.38–1.73	<0.001
75+	2.50	2.18–2.86	<0.001
Race and ethnicity (ref: White Non-Hispanic)	1		
Black Non-Hispanic	1.14	1.06–1.22	<0.001
Hispanic	0.82	0.73–0.92	<0.001
Other	0.8	0.71–0.90	<0.001
Hormone receptor status: positive vs negative	0.53	0.48–0.58	<0.001
AJCC clinical stage (ref: I)	1		
II	1.32	1.21–1.44	<0.001
III	2.53	2.31–2.78	<0.001
Comorbidity (ref: 0)	1		
1	1.22	1.13–1.31	<0.001
2+	1.82	1.63–2.04	<0.001
Chemo agent: single vs multiple	1.20	1.10–1.31	<0.001
Insurance (ref: Private)	1		
Medicaid	1.43	1.31–1.56	<0.001
Medicare	1.45	1.32–1.59	<0.001
Other government insurance	1.31	1.04–1.66	0.02
No insurance	1.45	1.25–1.68	<0.001
Income quartile (ref. 1st Quartile)	1		
2nd Quartile	0.92	0.85–1.01	0.07
3rd Quartile	0.85	0.78–0.93	<0.001
4th Quartile (richest)	0.76	0.70–0.82	<0.001
Facility Type (ref: Community Cancer Program (CCP))	1		
Comprehensive CCP	0.99	0.89–1.10	0.84
Academic	0.87	0.82–0.93	<0.001
Integrated Network	0.89	0.83–0.96	0.002

*CI* confidence interval, *CCP* Cancer Care Program.

Note: patients diagnosed in the last year (2022) did not have survival information.

### Sensitivity Analyses

Results from multivariable logistic regression and Cox proportional hazards models that included patients who died within one year of diagnosis were consistent with our primary analyses, suggesting that the exclusion of early deaths did not affect our findings (Supplementary Table 3, 4 and 5).

## Discussion

Our study highlights the persisting disparities in NACT patterns and outcomes among women with early stage HER2-positive BC, emphasizing the need to address them to improve overall treatment outcomes. We examined the predictors of NACT use and pCR, and the association between pCR and OS in a large cohort of patients. We observed a significantly increasing trend in NACT use from 18.6% in 2010 to 63.4% in 2022. Of note, patients with clinical stage III disease had a NACT use increase from 50.9% to 90.7%. Additionally, there was a steep increase in NACT use from 44.8% in 2020 to 60.9% in 2021. While the COVID pandemic may have contributed to these findings, the trend is likely a reflection of the current clinical practice guidelines. In particular, the mature results from the KATHERINE study which demonstrated a significant improvement in invasive disease-free survival with the use of adjuvant T-DM1 among patients with residual disease after NACT, have likely reinforced the role of NACT as an important component in the management of patients with early-stage HER2-positive BC^20–24^. Though multiple studies have reported on the trends in NACT in early-stage BC^13–17,25–28^, a few have assessed this in patients with HER2-positive disease^14,18,19,27,29^. Our study provides the most recent data showing the changing practice patterns with increasing use of chemotherapy for a disease that was historically aggressive and associated with poorer survival outcomes, while also highlighting ongoing disparities in care delivery.

Dual HER2 blockade, in combination with chemotherapy, has remained the standard of care in the management of high-risk early-stage HER2-positive BC. The American Society of Clinical Oncology (ASCO) guidelines recommend the use of dual HER2 blockade with trastuzumab and pertuzumab in combination with chemotherapy in the neoadjuvant setting as it significantly increases pCR rates^6^. However, disparities in the use of anti-HER2 therapy have been reported with Black and Hispanic patients being less likely to receive HER2-targeted therapies than White patients^30–34^. Reeder-Hayes et al. reported that Black women were 25% less likely to receive trastuzumab within one year of diagnosis than White women and this was especially pronounced among women with stage III disease^30^. More recent findings presented at the 2024 ASCO Quality Care Symposium noted that although Black and Hispanic patients remained less likely to receive HER2-targeted therapies than White patients, these disparities narrowed by 2018-2019, with no significant differences observed across racial and ethnic groups^31^.

pCR rates have steadily increased over time, reflecting the evolution of treatment regimens and their intensity. Despite these improvements, racial and ethnic disparities continue to be reflected in the rates of pCR. In our study, when stratified by race and ethnicity, we observed that Hispanic patients consistently had higher pCR rates across clinical stages, while Black patients had the lowest pCR rates. Higher tumor burden was associated with pCR rates as larger tumors and node positive disease were less likely to achieve a pCR. Since our multivariable model adjusted for clinical T and N stages, residual differences in pCR rates by race and ethnicity suggest that there are other plausible contributing factors. Overall, both Black and Hispanic patients are at higher risk of being diagnosed with more aggressive subtypes of BC, including HER2-positive disease^35,36^. However, we observed that Black patients were less likely to receive NACT and achieve pCR compared to the White patients in our population. This is similar to findings from the Chicago Multiethnic Epidemiologic Breast Cancer Cohort (ChiMEC) which reported that Black patients with early stage HER2-positive BC had lower odds of achieving pCR than White patients^37^. Additionally, they found that Black patients were more likely to have alterations in the MAPK pathway, identified as a possible resistance mechanism^37^. In addition to disease biology, systemic factors may also contribute to these findings. Black patients have more barriers to health care, present with more advanced disease, and have more comorbidities, influencing treatment decisions and outcomes^38,39^. Furthermore, prior studies have reported greater toxicity rates, suboptimal dosing and lower rates of treatment completion among Black patients being treated with systemic therapy for early-stage HER2-positive BC^30,40^. There is a need to not only address treatment access but to identify and improve factors that may contribute to poorer treatment outcomes among underrepresented groups.

Though Black and Hispanic women have a higher likelihood of not receiving guideline concordant BC treatment overall and by different subtypes^41^, we noted that Hispanic patients had higher odds of receiving NACT, but similar pCR rates when compared to White women. Another study using the NCDB found that overall, Hispanic patients had higher pCR rates but when stratified by BC subtypes, their pCR rates were similar to White patients with HER2-positive disease^42^. While current guidelines recommend the use of NACT in patients with high-risk node-negative or node positive disease, concerns remain about the receipt of guideline concordant care. A population-based cohort study of adult women with stage I-III triple negative or HER2-positive BC found that a substantial number of patients do not receive NACT as their first treatment, furthermore, the majority the patients who did not receive NACT, were not assessed by a medical oncologist prior to initial treatment^14^. Racial and ethnic disparities in BC treatment are multifactorial and require the use of culturally targeted interventions to narrow the gaps across different groups.

Our findings underscore the importance of adapting therapies based on treatment responses and showed that among patients with stage I-III HER2-positive BC, 3-year OS rates increased significantly overall, and particularly among those without a pCR. This improvement could be explained by the advancements in treatment strategies and NACT regimens, potentially leading to less residual disease. However, among patients with residual disease, Black patients persistently had the lowest 3-year OS rates from 2010 to 2018. ASCO guidelines recommend 14 cycles of adjuvant trastuzumab emtansine (T-DM1) among those with residual disease due to evidence showing an improvement in invasive disease-free survival and OS in this population^43,44^. Yet, despite these evidence-based recommendations, Black patients consistently experience poorer outcomes, suggesting that healthcare disparities may result from a combination of biological factors, treatment delivery and social determinants of health. There may be genetic or molecular differences in HER2-positive tumors between racial groups that affect their responsiveness to therapies such as T-DM1^37^. Other issues related to cancer care delivery that may impact these outcomes include adherence to guideline-concordant therapies, inadequate access to advanced therapies, and timely treatment initiation. Our findings show that tailoring adjuvant therapies and other personalized treatment approaches have enhanced the efficacy of treatment regimens, leading to improved OS even among patients who do not achieve a pCR^45,46^. Notably, among patients who achieved a pCR, 3-year OS rates were high across all racial and ethnic groups suggesting that achieving pCR may attenuate survival differences between these groups. These results point to the urgent need for continued efforts to ensure that all patients, regardless of race or ethnicity, receive timely, effective guideline-concordant treatments as achieving pCR could help mitigate disparities in long-term outcomes.

Notably, patients with HR-positive tumors were significantly less likely to receive NACT and to achieve pCR. These tumors have been reported to have higher CCND1 which encode for cyclin D1, associated with resistance to chemotherapy^47,48^. Furthermore, the presence of hormone receptors has consistently been associated with lower pCR rates relative to HR-negative tumors^49–51^. This is consistent with our findings where pCR rates in 2022 was 39% for HR-positive tumors and 64.6% for HR-negative tumors. Despite having lower rates of pCR, patients with HER2-positive, HR-positive tumors have good outcomes when treated with appropriate adjuvant therapies^12,49^. As expected, larger tumors and higher nodal stages were associated with increased odds of receiving NACT but were less likely to achieve pCR. This is similar to other studies that have reported that patients with lower clinical tumor stages and fewer lymph node involvement are more likely to achieve higher pCR rates^52,53^. These larger tumors may possess more aggressive disease biology and exhibit complete molecular profiles predisposing to chemotherapy and targeted therapy resistance^54^.

Patients with HER2-positive BC on Medicaid or Medicare were less likely to receive NACT and when they did, they were less likely to achieve a pCR. While there was no significant difference in NACT use among patients with no insurance compared to those with private insurance, uninsured patients were still less likely to have a pCR. These findings are consistent with studies that have evaluated disparities among patients with BC^6,55–57^. Socioeconomic factors play a major role in the access to, and the quality of cancer care that patients receive. Uninsured patients and those insured through Medicaid or Medicare may experience delays in treatment initiation, lower adherence to prescribed therapies and more limited access to specialized oncology care, which contribute to the lower odds of receiving NACT and having a pCR^58,59^. This illustrates a need for policy interventions to ensure that all patients have equal access to high-quality cancer care as timely and appropriate administration of NACT is necessary for improving pCR rates and survival outcomes.

In line with other studies^60–62^, our results showed that patients who achieved a pCR had improved survival outcomes compared to those that did not. However, even after adjusting for pCR, survival differences persisted by other socioeconomic status and clinical characteristics. Hispanic patients and those in higher income quartiles had higher survival. On the other hand, higher clinical stage, presence of comorbidities and not having private insurance were associated with poorer survival. These findings specifically highlight the negative impact of social determinants of health on outcomes in HER2-positive BC. Systematic and multi-layered efforts are needed to study treatment-related disparities and proffer sustainable solutions that will lead to superior health outcomes for all, regardless of their prevailing socioeconomic situation.

Our study has several limitations. NCDB does not contain detailed information regarding systemic therapy regimens or number of cycles administered. It also lacks information on the amount of residual disease and genomic information; thus, we were unable to investigate differences across different subpopulations and its impact on outcomes. The NCDB database does not contain information on social support systems, health literacy, mental health or neighborhood-level factors like pollution, neighborhood poverty and food insecurity, hence, we could not conduct more detailed analyses on these social and behavioral factors to understand how they are associated with NACT use and outcomes. Although we controlled for possible confounders, there is still potential for residual confounders such as insurance-related delays and patient preferences. Data collection for the NCDB is performed by trained data abstractors, but coding errors and misclassification bias cannot be completely excluded. Furthermore, while the NCDB is a large, comprehensive database, it relies on the reporting practices of individual hospitals which may vary and affect the accuracy of the information. Also, despite covering over 80% of BC cases treated in the US, generalizability remains limited because the data are hospital-based. Notwithstanding these limitations, our findings highlight important trends and factors predicting NACT use and pCR in a large cohort of patients with localized HER2-positive BC.

In conclusion, this work revealed significant trends and disparities in the use of NACT and pCR rates among patients with early-stage HER2-positive BC. We observed an increasing trend in NACT use and pCR rates over the last decade, alongside improvements in OS, especially among patients who did not achieve a pCR. Notably, the greatest improvements in OS among those without pCR was observed in patients with HR-negative disease, likely reflecting the increased use of effective post-neoadjuvant strategies like T-DM1. While patients with HR-positive tumors generally have better long-term outcomes, possibly due to the benefits of endocrine therapy, the survival gains in those with HR-negative disease demonstrates the importance of intensifying systemic treatment in this population. Despite improved outcomes, racial and ethnic disparities persist, with Black patients being less likely to receive NACT and achieve pCR, while Hispanic patients were more likely to receive NACT but had similar pCR rates as the White patients. These disparities underscore the need for equitable access to care and for future research focused on understanding how racial and ethnic factors influence treatment access and outcomes. Addressing these disparities will be essential to ensuring that all patients, particularly those from minoritized populations, benefit equally from the ongoing advancements in care. Lastly, as ongoing clinical trials explore de-escalation strategies to reduce treatment-related toxicities for patients^63,64^, efforts should be made to ensure that these approaches are implemented equitably to help close the persisting racial and ethnic gaps in HER2-positive BC care and outcomes.

## Methods

### Study design and population

Patients aged 18 years or older diagnosed with clinical stage I-III HER2-positive BC between 2010 and 2022 who underwent surgery and received chemotherapy were selected from the National Cancer Database (NCDB) using a retrospective cohort design. The study population was limited to patients who did not die within one year after diagnosis to reduce the heterogeneity in the cohort and minimize the risk of inadvertently including individuals with undiagnosed stage IV disease at presentation (Supplementary Table 1). Pathologic complete response (pCR) was defined as the absence of residual invasive cancer on the resected breast and lymph node specimen after NACT (ypT0/Tis ypN0). Using the pathologic tumor size and regional lymph nodes information available in the database, patients with ypT0 or ypTis (ductal carcinoma in situ) and ypN0 were categorized as having a pCR while those with other pathologic T and N categories had residual disease.

### Data source

The NCDB is the largest hospital-based clinical cancer registry that collects information from over 1500 Commission on Cancer (CoC)-accredited facilities, covering 30% of hospitals and more than 80% of all BC cases^65^. It is a national comprehensive clinical database jointly sponsored by the American College of Surgeons and the American Cancer Society^66^. Data collected include sociodemographic, clinical, treatment-related and follow-up information^65^. The study was reviewed by the Institutional Review Board (IRB) of the University of Texas MD Anderson Cancer Center and was determined to be exempt from further IRB review. Because a large, de-identified database was used for the research, informed consent was not required. The study was conducted in accordance with the principles of the Declaration of Helsinki.

### Sociodemographic and clinical variables

Variables selected for inclusion in the study were age (18–49, 50–59, 60–64, 65–69, ≥75), race and ethnicity (Black, Hispanic, non-Hispanic White, other), Hormone receptor status (negative, positive), American Joint Committee on Cancer (AJCC) clinical stage (I-III), AJCC clinical tumor stage (T1-T4), AJCC clinical nodal status (N0-N3), Charlson Deyo comorbidity score (0, 1, 2+), chemotherapy agent (single or multiple), insurance type (Private, Medicaid, Medicare, other government insurance or no insurance), education quartile, income quartile, area of residence (metropolitan, urban or rural), region (South, Northeast, Midwest or West) and facility type (Community cancer program [CCP], Comprehensive CCP, Academic or Integrated network).

### Statistical analysis

Baseline characteristics were described by chemotherapy receipt modality (neoadjuvant or adjuvant) using frequencies and Pearson’s chi square statistics. The Cochran-Armitage test for time trends was used to analyze the trends in NACT use, pCR rates and 3-year OS among patients with HER2-positive BC from 2010 to 2022. Trends in NACT use were assessed both overall and stratified by AJCC clinical stage, while trends in pCR rates among HER2-positive BC patients who underwent NACT were explored overall and stratified by hormone receptor (HR) status. Rates of pCR were evaluated by race and ethnicity, HR status, and clinical stage. Furthermore, trends in 3-year OS were assessed by both HR and pCR status. Among patients with residual disease, 3-year OS was estimated by year of diagnosis and race and ethnicity. Multivariable logistic regression models were used to examine factors associated with NACT use and pCR among patients who received chemotherapy and NACT, respectively. Additionally, multivariable Cox proportional hazards model with propensity score (PS) adjustment was used to examine the association between pCR and OS. Logistic regression was used to estimate the propensity score, and this was then included as a covariate in the multivariable cox proportional hazards model. OS was defined as the time from BC diagnosis to either the date of death (event) or the last follow-up (censoring). One-to-one propensity score matching was performed by exact year of diagnosis, HR-status and clinical stage, and 3-year OS was examined based on matched pairs (*n* = 26,870 pairs), comparing patients who achieved a pCR to those that did not. The variables included in the final models were identified based on clinical and statistical significance and included year of diagnosis, age at diagnosis, race and ethnicity, hormone receptor status, clinical tumor size, clinical nodal stage, comorbidity score, use of single or multiple chemotherapy agents, insurance type, income quartiles, region, area of residence and facility type. To evaluate the robustness of our findings, we performed sensitivity analyses that included patients who died within one year of diagnosis. Here, the multivariable logistic regression and cox proportional hazards regression analyses were repeated in the cohort that included those who died within one year of diagnosis. All analyses were conducted using SAS, version 9.4 (SAS Institute, Cary NC), with statistical significance determined by a 2-sided p-value of less than 0.05.

## Supplementary information

Supplementary Information

## Data Availability

The data that support the findings of this study are available from the American College of Surgeons’ National Cancer Database (NCDB) at https://www.facs.org/quality-programs/cancer-programs/national-cancer-database but restrictions apply to the availability of these data. Data are however available from the authors upon reasonable request and with permission from the NCDB. The underlying code for this study is not publicly available but may be made available to qualified researchers on reasonable request from the corresponding author.

## Code availability

The underlying code for this study is not publicly available but may be made available to qualified researchers on reasonable request from the corresponding author.
