# Supplementary material for: Racial and ethnic disparities in neoadjuvant chemotherapy patterns and outcomes in early-stage HER2-positive breast cancer
Source: NPJ Breast Cancer. 2025 Dec 5;11:138. doi: 10.1038/s41523-025-00854-4 (PMC12680642; doi:10.1038/s41523-025-00854-4)
Supplement: Supplementary file 1 — Supplementary Information [file 41523_2025_854_MOESM1_ESM.pdf]

Supplementary Table 1. Cohort selection

Step	Cohort Selection Criteria	Total N
1	Patients with breast cancer in NCDB.	4,231,162
2	Year of diagnosis 2010-2022.	3,135,652
3	Clinical stage I-III.	2,123,125
4	Patients did not die within 1 year after diagnosis.	2,086,867
5	Patients had surgery of lumpectomy or mastectomy.	1,982,090
6	Chemotherapy use status was known (no chemo or had chemo within +/-6 months of diagnosis) (all subtypes).	1,900,130
7	Subtype as HER2+.	238,593
Cohort 1	Included patients with HER2+ breast cancer who had chemotherapy.	195,023
Cohort 2	Included patients with HER2+ breast cancer who had neoadjuvant chemotherapy.	73,627

Supplementary Table 2. Baseline characteristics by pathologic complete response (pCR) among patients with HER2+ BC who underwent neoadjuvant chemotherapy (NACT) (n=73,627)

**Covariates**	**Total N (Column %)**	**no pCR N (Row %)**	**Yes pCR N (Row %)**	**P**
Total	73627 (100)	43035 (58.5)	30592 (41.5)	
Age at diagnosis				
18-49	27377 (37.2)	15449 (56.4)	11928 (43.6)	<0.001
50-59	21504 (29.2)	12131 (56.4)	9373 (43.6)	
60-64	9290 (12.6)	5495 (59.1)	3795 (40.9)	
65-74	11651 (15.8)	7293 (62.6)	4358 (37.4)	
75+	3805 (5.2)	2667 (70.1)	1138 (29.9)	
Race and ethnicity				
Black	9342 (12.7)	5751 (61.6)	3591 (38.4)	<0.001
Hispanic	6566 (8.9)	3682 (56.1)	2884 (43.9)	
White Non-Hispanic	51574 (70)	30265 (58.7)	21309 (41.3)	
Other	6145 (8.3)	3337 (54.3)	2808 (45.7)	
Hormone receptor status				
Negative	24394 (33.1)	10900 (44.7)	13494 (55.3)	<0.001
Positive	49233 (66.9)	32135 (65.3)	17098 (34.7)	
AJCC clinical stage				
I	22618 (30.7)	14101 (62.3)	8517 (37.7)	<0.001
II	36575 (49.7)	20184 (55.2)	16391 (44.8)	
III	14434 (19.6)	8750 (60.6)	5684 (39.4)	
AJCC clinical tumor stage				
T0	114 (0.2)	65 (57)	49 (43)	
T1	16231 (22)	9280 (57.2)	6951 (42.8)	<0.001
T2	40379 (54.8)	23394 (57.9)	16985 (42.1)	
T3	10811 (14.7)	6442 (59.6)	4369 (40.4)	
T4	5859 (8)	3703 (63.2)	2156 (36.8)	
Unknown	233 (0.3)	151 (64.8)	82 (35.2)	
AJCC clinical nodal status				
N0	41437 (56.3)	23811 (57.5)	17626 (42.5)	<0.001
N1	25908 (35.2)	15401 (59.4)	10507 (40.6)	
N2	3345 (4.5)	2058 (61.5)	1287 (38.5)	
N3	2683 (3.6)	1569 (58.5)	1114 (41.5)	
Unknown	254 (0.3)	196 (77.2)	58 (22.8)	
Comorbidity				
0	63924 (86.8)	37092 (58)	26832 (42)	<0.001

1	7525 (10.2)	4550 (60.5)	2975 (39.5)	
2+	2178 (3)	1393 (64)	785 (36)	
Chemo agent				
Multiple	65275 (88.7)	37813 (57.9)	27462 (42.1)	<0.001
Single	7013 (9.5)	4356 (62.1)	2657 (37.9)	
Unknown	1339 (1.8)	866 (64.7)	473 (35.3)	
Insurance				
Private	47287 (64.2)	26638 (56.3)	20649 (43.7)	<0.001
Medicaid	7426 (10.1)	4410 (59.4)	3016 (40.6)	
Medicare	15191 (20.6)	9727 (64)	5464 (36)	
Other government insurance	1020 (1.4)	584 (57.3)	436 (42.7)	
No insurance	1939 (2.6)	1207 (62.2)	732 (37.8)	
Unknown	764 (1)	469 (61.4)	295 (38.6)	
Education quartile				
1 least educated	11527 (15.7)	6875 (59.6)	4652 (40.4)	<0.001
2	14903 (20.2)	8907 (59.8)	5996 (40.2)	
3	17973 (24.4)	10596 (59)	7377 (41)	
4	18397 (25)	10467 (56.9)	7930 (43.1)	
Unknown	10827 (14.7)	6190 (57.2)	4637 (42.8)	
Income quartile				
1st Quartile	9307 (12.6)	5734 (61.6)	3573 (38.4)	<0.001
2nd Quartile	12227 (16.6)	7251 (59.3)	4976 (40.7)	
3rd Quartile	14913 (20.3)	8734 (58.6)	6179 (41.4)	
4th Quartile (richest)	26341 (35.8)	15118 (57.4)	11223 (42.6)	
Unknown	10839 (14.7)	6198 (57.2)	4641 (42.8)	
Area of residence[a]				
Metro	61016 (82.9)	35577 (58.3)	25439 (41.7)	0.004
Urban	8718 (11.8)	5165 (59.2)	3553 (40.8)	
Rural	1392 (1.9)	833 (59.8)	559 (40.2)	
Unknown	2501 (3.4)	1460 (58.4)	1041 (41.6)	
Region				
South	24288 (33)	14654 (60.3)	9634 (39.7)	<0.001
Northeast	11188 (15.2)	6672 (59.6)	4516 (40.4)	
Midwest	15974 (21.7)	9228 (57.8)	6746 (42.2)	
West	11952 (16.2)	6789 (56.8)	5163 (43.2)	
Unknown	10225 (13.9)	5692 (55.7)	4533 (44.3)	
Facility Type				
Community Cancer Program (CCP)	24640 (33.5)	14850 (60.3)	9790 (39.7)	<0.001
Comprehensive CCP	4049 (5.5)	2535 (62.6)	1514 (37.4)	

Academic	20511 (27.9)	11648 (56.8)	8863 (43.2)	
Integrated Network	14202 (19.3)	8310 (58.5)	5892 (41.5)	
Unknown	10225 (13.9)	5692 (55.7)	4533 (44.3)	

AJCC, American Joint Committee on Cancer. CCP, Cancer Care Program. pCR, pathologic complete response.
[a] Estimated by matching the state/county Federal Information Processing Standards code of the patient at diagnosis to 2013 data published by the US Department of Agriculture Economic Research Service. Metropolitan counties are defined as having a population size of the metropolitan area greater than 250 000. Urban counties are defined as non-metropolitan with a population size of at least 2500. Rural counties have a population of fewer than 2500.

Supplementary Table 3. Multivariable logistic regression model for receipt of neoadjuvant chemotherapy among patients with HER2+ BC who underwent chemotherapy, excluding those who died in 1 year (n=195,023) or including those who died in 1 year (n=196,181)

	**Excluding those who died in 1 year (n=195,023)**			**Including those who died in 1 year (n=196,181)**		
**Covariates**	**Odds Ratio**	**95% CI**	**P**	**Odds Ratio**	**95% CI**	**P**
Year of diagnosis (ref: 2010-2013)	1			1		
2014-2017	2.64	2.56-2.71	<0.001	2.63	2.56-2.71	<0.001
2018-2022	5.36	5.20-5.52	<0.001	5.35	5.20-5.51	<0.001
Age at diagnosis (ref: 18-49)	1			1		
50-59	0.87	0.85-0.90	<0.001	0.87	0.84-0.90	<0.001
60-64	0.8	0.77-0.83	<0.001	0.79	0.77-0.82	<0.001
65-74	0.75	0.72-0.79	<0.001	0.75	0.71-0.78	<0.001
75+	0.78	0.74-0.83	<0.001	0.77	0.73-0.82	<0.001
Race and ethnicity (ref: White Non-Hispanic)	1			1		
Black Non-Hispanic	0.96	0.93-0.99	0.016	0.96	0.93-0.99	0.022
Hispanic	1.11	1.06-1.15	<0.001	1.11	1.06-1.16	<0.001
Other	1.04	1.00-1.08	0.07	1.04	1.00-1.09	0.043
Hormone receptor status: positive vs negative	0.82	0.80-0.84	<0.001	0.82	0.81-0.84	<0.001
AJCC clinical tumor size (ref: T1)	1			1		
T2	4.07	3.97-4.17	<0.001	4.06	3.96-4.16	<0.001
T3	5.35	5.15-5.56	<0.001	5.36	5.15-5.57	<0.001
T4	8.19	7.77-8.64	<0.001	8.20	7.77-8.65	<0.001
AJCC clinical nodal stage (ref: N0)	1			1		
N1	1.14	1.11-1.17	<0.001	1.14	1.11-1.17	<0.001
N2	1.23	1.16-1.30	<0.001	1.23	1.16-1.30	<0.001
N3	1.12	1.04-1.19	0.001	1.11	1.04-1.19	0.001
Comorbidity (ref: 0)	1			1		
1	0.88	0.85-0.91	<0.001	0.88	0.85-0.91	<0.001
2+	0.84	0.79-0.89	<0.001	0.83	0.78-0.88	<0.001

Chemo agent: single vs multiple	0.39	0.38-0.40	<0.001	0.39	0.38-0.40	<0.001
Insurance (ref: Private)	1			1		
Medicaid	0.94	0.91-0.98	0.001	0.94	0.91-0.98	0.002
Medicare	0.92	0.88-0.96	<0.001	0.92	0.88-0.95	<0.001
Other government insurance	1	0.91-1.10	0.98	1.00	0.91-1.09	0.97
No insurance	0.99	0.93-1.07	0.89	1.00	0.93-1.07	0.92
Income quartile (ref: 1st Quartile)	1			1		
2nd Quartile	1.04	1.00-1.08	0.07	1.04	1.00-1.08	0.06
3rd Quartile	1.09	1.05-1.13	<0.001	1.09	1.05-1.13	<0.001
4th Quartile (richest)	1.11	1.07-1.15	<0.001	1.11	1.07-1.16	<0.001
Area of residence (ref: Metro)	1			1		
Urban	0.92	0.89-0.95	<0.001	0.92	0.89-0.95	<0.001
Rural	0.92	0.85-0.99	0.032	0.92	0.85-0.99	0.029
Region (ref: South)	1			1		
Northeast	0.88	0.85-0.91	<0.001	0.88	0.85-0.91	<0.001
Midwest	1.05	1.02-1.08	<0.001	1.05	1.02-1.08	<0.001
West	1.04	1.01-1.08	0.012	1.05	1.01-1.08	0.008
Facility Type (ref: Community Cancer Program (CCP))	1			1		
Comprehensive CCP	0.92	0.88-0.96	<0.001	0.92	0.87-0.96	<0.001
Academic	1.08	1.05-1.11	<0.001	1.08	1.05-1.11	<0.001
Integrated Network	1.12	1.08-1.15	<0.001	1.11	1.08-1.15	<0.001

CI, confidence interval; CCP, Cancer Care Program.

Supplementary Table 4. Multivariable logistic regression model for pathologic complete response (pCR) among patients with HER2+ BC who had neoadjuvant chemotherapy, excluding those who died in 1 year (n=73,627) or including those who died in 1 year (n=73,847)

	Excluding those who died in 1 year (n=73,627)			Including those who died in 1 year (n=73,847)		
**Covariates**	**Odds Ratio**	**95% CI**	**P**	**Odds Ratio**	**95% CI**	**P**
Year of diagnosis (ref: 2010-2013)	1			1		
2014-2017	1.68	1.59-1.76	<0.001	1.68	1.59-1.77	<0.001
2018-2022	2.37	2.26-2.49	<0.001	2.38	2.27-2.50	<0.001
Age at diagnosis (ref: 18-49)	1			1		
50-59	0.97	0.93-1.01	0.16	0.97	0.93-1.01	0.17
60-64	0.87	0.82-0.91	<0.001	0.87	0.82-0.91	<0.001
65-74	0.78	0.73-0.83	<0.001	0.77	0.72-0.83	<0.001
75+	0.56	0.51-0.62	<0.001	0.56	0.51-0.62	<0.001
Race and ethnicity (ref: White Non-Hispanic)	1			1		
Black Non-Hispanic	0.86	0.82-0.90	<0.001	0.86	0.82-0.90	<0.001
Hispanic	1.05	1.00-1.11	0.07	1.06	1.00-1.12	0.06
Other	1.08	1.02-1.14	0.011	1.07	1.02-1.14	0.013
Hormone receptor status: positive vs negative	0.39	0.38-0.40	<0.001	0.39	0.38-0.41	<0.001
AJCC clinical tumor size (ref: T1)	1			1		
T2	0.94	0.90-0.98	0.002	0.94	0.91-0.98	0.002
T3	0.87	0.83-0.92	<0.001	0.87	0.82-0.91	<0.001
T4	0.81	0.76-0.87	<0.001	0.81	0.76-0.87	<0.001
AJCC clinical nodal stage (ref: N0)	1			1		
N1	0.92	0.89-0.95	<0.001	0.92	0.89-0.95	<0.001
N2	0.91	0.84-0.98	0.014	0.91	0.84-0.98	0.011
N3	0.94	0.86-1.02	0.15	0.94	0.86-1.02	0.13
Comorbidity (ref: 0)	1			1		
1	0.94	0.90-0.99	0.022	0.94	0.90-0.99	0.022
2+	0.84	0.76-0.92	<0.001	0.83	0.76-0.91	<0.001
Chemo agent: single vs multiple	0.86	0.82-0.91	<0.001	0.86	0.81-0.91	<0.001
Insurance (ref: Private)	1			1		
Medicaid	0.87	0.83-0.92	<0.001	0.87	0.83-0.92	<0.001
Medicare	0.90	0.85-0.96	<0.001	0.90	0.85-0.96	0.001
Other government insurance	0.98	0.86-1.12	0.81	0.98	0.86-1.12	0.79
No insurance	0.80	0.73-0.89	<0.001	0.80	0.72-0.88	<0.001
Income quartile (ref: 1st Quartile)	1			1		

2nd Quartile	1.07	1.01-1.13	0.024	1.07	1.01-1.13	0.022
3rd Quartile	1.10	1.04-1.16	0.001	1.10	1.04-1.16	<0.001
4th Quartile (richest)	1.10	1.04-1.16	<0.001	1.10	1.04-1.16	<0.001
Region (ref: South)	1			1		
Northeast	0.98	0.94-1.03	0.53	0.98	0.94-1.03	0.53
Midwest	1.09	1.04-1.14	<0.001	1.09	1.04-1.14	<0.001
West	1.12	1.07-1.18	<0.001	1.12	1.07-1.18	<0.001
Facility Type (ref: Community Cancer Program (CCP))	1			1		
Comprehensive CCP	0.91	0.85-0.98	0.012	0.91	0.85-0.98	0.01
Academic	1.15	1.10-1.20	<0.001	1.15	1.10-1.20	<0.001
Integrated Network	1.06	1.02-1.11	0.006	1.06	1.02-1.11	0.006

CI, confidence interval; CCP, Cancer Care Program.

Supplementary Table 5. Multivariable Cox proportional hazards model for the association of pathologic complete response (pCR) with overall survival among patients with HER2+ BC who had neoadjuvant chemotherapy with propensity score adjustment excluding those who died within 1 year (n=64,203) or including those who died within 1 year (n=64,424)

Note: patients diagnosed in the last year (2022) did not have mortality information.

	Excluding those who died within 1 year (n=64,203)			Including those who died within 1 year (n=64,424)		
**Covariates**	**Hazard Ratio**	**95% CI**	**P**	**Hazard Ratio**	**95% CI**	**P**
pCR: yes vs no	0.45	0.42-0.48	<0.001	0.42	0.39 - 0.45	<0.001
Age at diagnosis (ref: 18-49)	1			1		
50-59	1.25	1.15-1.35	<0.001	1.26	1.16 - 1.36	<0.001
60-64	1.32	1.20-1.46	<0.001	1.38	1.25 - 1.52	<0.001
65-74	1.55	1.38-1.73	<0.001	1.66	1.48 - 1.86	<0.001
75+	2.50	2.18-2.86	<0.001	2.96	2.61 - 3.37	<0.001
Race and ethnicity (ref: White Non-Hispanic)	1			1		
Black Non-Hispanic	1.14	1.06-1.22	<0.001	1.20	1.12 - 1.30	<0.001
Hispanic	0.82	0.73-0.92	<0.001	0.77	0.69 - 0.87	<0.001
Other	0.8	0.71-0.90	<0.001	0.78	0.69 - 0.88	<0.001
Hormone receptor status: positive vs negative	0.53	0.48-0.58	<0.001	0.72	0.68 - 0.76	<0.001
AJCC clinical stage (ref: I)	1			1		
II	1.32	1.21-1.44	<0.001	1.45	1.33 - 1.58	<0.001
III	2.53	2.31-2.78	<0.001	2.98	2.73 - 3.26	<0.001
Comorbidity (ref: 0)	1					
1	1.22	1.13-1.31	<0.001	1.23	1.14 - 1.33	<0.001
2+	1.82	1.63-2.04	<0.001	1.87	1.67 - 2.10	<0.001
Chemo agent: single vs multiple	1.20	1.10-1.31	<0.001	1.26	1.15 - 1.37	<0.001
Insurance (ref: Private)	1			1		
Medicaid	1.43	1.31-1.56	<0.001	1.53	1.40 - 1.67	<0.001
Medicare	1.45	1.32-1.59	<0.001	1.52	1.39 - 1.67	<0.001
Other government insurance	1.31	1.04-1.66	0.02	1.35	1.07 - 1.71	0.012
No insurance	1.45	1.25-1.68	<0.001	1.63	1.40 - 1.89	<0.001
Income quartile (ref: 1st Quartile)	1			1		
2nd Quartile	0.92	0.85-1.01	0.07	0.90	0.83 - 0.98	0.021
3rd Quartile	0.85	0.78-0.93	<0.001	0.82	0.75 - 0.89	<0.001
4th Quartile (richest)	0.76	0.70-0.82	<0.001	0.72	0.67 - 0.79	<0.001
Facility Type (ref: Community Cancer Program (CCP))	1			1		
Comprehensive CCP	0.99	0.89-1.10	0.84	0.99	0.89 - 1.11	0.92

Academic	0.87	0.82-0.93	<0.001	0.82	0.77 - 0.88	<0.001
Integrated Network	0.89	0.83-0.96	0.002	0.85	0.79 - 0.92	<0.001

CI, confidence interval; CCP, Cancer Care Program.
